# Allied health – 3009. Comparative assessment of dental caries experience, oral hygiene status, gingival health status, salivary *Streptococcus* mutans count and *Lactabacillus* count between asthmatic and non-asthmatic children aged between 5-12 years in Davangere City, Karnataka, India

**DOI:** 10.1186/1939-4551-6-S1-P185

**Published:** 2013-04-23

**Authors:** Usha Venkatesh

**Affiliations:** 1 Public Health Dentistry, Bapuji Dental College and Hospital, Davanagere, India

## Background

To assess and compare the caries experience, oral hygiene status, gingival health status, salivary *Streptococcus mutans* count and *Lactobacillus* count between asthmatic children and a healthy control group.

## Methods

A cross sectional comparative study was conducted on 5-12 years old, 40 asthmatic and 62 non-asthmatic subjects in Davanagere city. Selection criteria – a) children with diagnosis of asthma as diagnosed by lung function test by the medical officer of the asthma care centre, Bapuji Hospital, Davanagere, who were currently on asthmatic medication belonging to 5-12 year age group residing in Davanagere city. b) The non-asthmatic status of selected controls was ascertained by asking certain relevant questions to the children and their parents which can elicit the presence of symptoms of asthma. Cases and controls were matched for gender, age, socio-economic status, diet and oral hygiene practices. Relevant and required information regarding demographic characteristics and asthmatic status was obtained. DMFT index and def index were used to assess the dental caries status. Oral Hygiene Simplified Index (OHI-S) and Gingival Index were used to assess the oral hygiene status and gingival status respectively. Paraffin stimulated saliva sample was collected from both asthmatic and non-asthmatic subjects for microbiological analysis.

## Results

A significant association was found between asthmatic status and dental caries (p<0.05). There were no significant differences in gingival status and oral hygiene status between asthmatic and non-asthmatic children. The number of S.mutans colonies was significantly different between the two groups (94.1 ± 60 colonies in the study group versus 57 ± 30.5 colonies in the control group; p<0.001).

## Conclusions

Asthmatic children had higher caries experience and it increased with duration of asthma. The frequency, duration and type of medication for asthma were not found to have any significant effect. Salivary S.mutans was high in asthmatic children where as salivary Lactobacillus was similar in both the groups.

